# Endovascular versus Surgical Lower Extremity Revascularization among Patients with Chronic Kidney Disease

**DOI:** 10.1155/2023/5586060

**Published:** 2023-12-16

**Authors:** Qingzheng Chen, Jialin Han, Gomathy Parvathinathan, Elsie Ross, Margaret R. Stedman, Tara I. Chang

**Affiliations:** ^1^Stanford University, Division of Nephrology, Department of Medicine, 3180 Porter Drive, Stanford, CA 94304, USA; ^2^Stanford University, Division of Vascular Surgery, Department of Surgery, 300 Pasteur Drive Room M121, Stanford, CA 94305, USA; ^3^Stanford University, Division of Biomedical Informatics Research, Department of Medicine, 300 Pasteur Drive Room M121, Stanford, CA 94305, USA; ^4^UC San Diego School of Medicine, Department of Surgery, Division of Vascular Surgery, La Jolla, CA 92037, USA

## Abstract

**Introduction:**

Patients with chronic kidney disease (CKD) have a high prevalence of peripheral artery disease. How best to manage lower extremity peripheral artery disease remains unclear in this patient population. We therefore sought to compare the outcomes after endovascular versus surgical lower extremity revascularization among patients with CKD.

**Methods:**

We used data from Optum's de-identifed Clinformatics® Data Mart Database, a nationwide database of commercially insured persons in the United States to study patients with CKD who underwent lower extremity endovascular or surgical revascularization. We used inverse probability of treatment weighting to balance covariates. We employed proportional hazard regression to study the primary outcome of major adverse limb events (MALE), defined as a repeat revascularization or amputation. We also studied each of these events separately and death from any cause.

**Results:**

In our cohort, 60,057 patients underwent endovascular revascularization and 9,338 patients underwent surgical revascularization. Endovascular revascularization compared with surgical revascularization was associated with a higher adjusted hazard of MALE (hazard ratio (HR) 1.52; 95% confidence interval (CI) 1.46–1.59). Endovascular revascularization was also associated with a higher adjusted hazard of repeat revascularization (HR 1.65; 95% CI 1.57–1.72) but a lower adjusted risk of amputation (HR 0.71; CI 0.73–0.89). Patients undergoing endovascular revascularization also had a lower adjusted hazard for death from any cause (0.85; CI 0.82–0.88).

**Conclusions:**

In this analysis of patients with CKD undergoing lower extremity revascularization, an endovascular approach was associated with a higher rate of repeated revascularization but a lower risk of subsequent amputation and death compared with surgical revascularization. Multiple factors must be considered when counseling patients with CKD, who have a high burden of comorbid conditions. Clinical trials should include more patients with kidney disease, who are often otherwise excluded from participation, to better understand the most effective treatment strategies for this vulnerable patient population.

## 1. Introduction

Patients with chronic kidney disease (CKD) have a high prevalence of peripheral artery disease (PAD), and patients with both CKD and PAD have a significantly higher mortality rate than patients with either disease alone [1–3]. The higher rates of concomitant CKD and PAD may be related to shared traditional risk factors such as hypertension, hyperlipidemia, and diabetes as well as to the fact that CKD confers a set of unique risk factors that can lead to progression of PAD, including chronic inflammation, hypoalbuminemia, uremia, and disorders of mineral metabolism that can accelerate vascular calcification [4, 5]. The progression of PAD can ultimately lead to a need for lower extremity revascularization to re-establish blood flow and prevent limb loss or lifestyle-limiting disease.

Lower extremity revascularization has evolved substantially in the last 20 years and can be achieved using endovascular or surgical approaches. Prior trials comparing endovascular and surgical approaches have specifically excluded patients with moderate-to-advanced CKD, leaving an evidence gap regarding the best approach for treating this population [6]. Understanding optimal revascularization approaches in patients with CKD patients is of particular importance due to the aggressive nature of atherosclerotic disease in this patient population, which often requires a multivessel surgical approach that can increase the risk of operative and perioperative morbidity and mortality. Thus, less invasive endovascular approaches may be favored [7, 8]. We therefore sought to compare outcomes after endovascular versus surgical lower extremity revascularization among patients with CKD, using Optum's de-identifed Clinformatics® Data Mart Database, a nationwide database of commercially insured persons in the United States.

## 2. Methods

### 2.1. Data Source

We collected data from Optum's de-identifed Clinformatics® Data Mart Database (CDM), a database comprised of administrative health claims for members of large national commercial and Medicare Advantage health plans. Access to the data is controlled by Optum, and inquiries about data access should be directed to Optum. These administrative claims are submitted for payment by providers and pharmacies, and are verified, adjudicated, adjusted, and deidentified prior to inclusion in the CDM. Data are included for only those covered lives with medical and prescription drug coverage to enable users to evaluate the claims related to the complete healthcare experience. In addition, CDM includes results for outpatient lab tests processed by large national laboratory vendors under contract with the managed care organization. The population is geographically diverse, spanning all 50 states.

### 2.2. Study Population

Our cohort included all patients who underwent lower extremity revascularization between January 1, 2013, and January 31, 2021, who were at least 18 years of age at the time of the procedure. We identified endovascular revascularization using International Classification of Diseases Ninth Edition (ICD-9) procedure codes 38.18, 39.50, and 39.90; Tenth Edition (ICD-10) procedure codes (Supplemental Table 1); and Current Procedural Terminology (CPT) codes 35302−06, 35331, 35381, 35452, 35454, 35456, 35459, 35470, 35472−74, 35481–35483, 35485, 35491–35493, 35495, 37184−89, 37205−08, and 37220–37239 [9, 10]. Surgical revascularization was identified using ICD-9 procedure codes 39.25, 39.29, 38.08, 38.38, 38.48, 39.49, 39.56, 39.57, and 39.58; ICD-10 procedure codes (Supplemental Table 2); and CPT codes 35226, 35256, 35286, 35351, 35355, 35361, 35363, 35371-72, 35521, 35533, 35537−41, 35546, 35548-49, 35551, 35556, 35558, 35563, 35565-66, 35571, 35582-83, 35585, 35587, 35621, 35623, 35637-38, 35641,35646-47, 35651, 35654, 35656, 35661, 35663, 35665-66, 35671, 35700, 35721, 35741, 35876, 35879, 35881, 35883, and 35884.

Our exposure of interest was lower extremity revascularization. We defined the index day as the day of revascularization. We excluded patients without at least one year of continuous enrollment prior to the index date, because we ascertained comorbid conditions based on claims accrued during this interval. Given that the focus of this analysis is on patients with CKD, we excluded patients without evidence of CKD in the one year prior to the index date. We defined CKD by the presence of either a qualifying ICD-9 or ICD-10 code or a qualifying laboratory result. We used the same ICD-9 or ICD-10 code as is used by the Centers for Disease Control CKD Surveillance program [11] to define CKD. All laboratory values in the Optum database are collected from the outpatient setting. Patients were categorized as having CKD if they had an estimated glomerular filtration rate <60 mL/min per 1.73 m [2] (calculated using the 2021 CKD-EPI creatinine refit equation without race [12]) or albuminuria, defined as an urinary albumin-to-creatinine ratio >30 mg/g or a urinary protein-to-creatinine ratio >150 mg/g. Patients with end-stage kidney disease were included in the present analysis.

**Figure 1:**
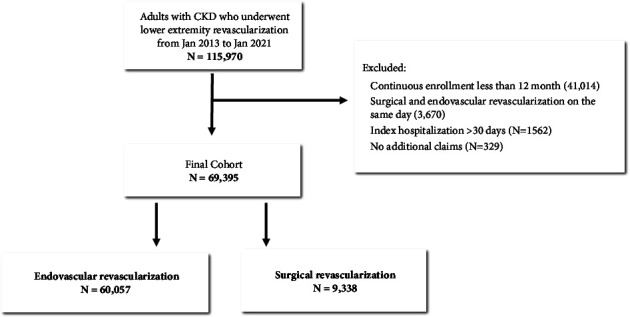
Cohort assembly of adults with chronic kidney disease (CKD) who underwent lower extremity revascularization between January 2013 and January 2021.

We additionally excluded patients who underwent surgical and endovascular revascularization on the same day, patients with exceptionally long index hospitalization (i.e., >30 days), and patients with no additional claims after the index date (Figure 1).

### 2.3. Outcomes

The main outcome of interest was a major adverse limb event (MALE), defined as a lower extremity amputation (ascertained using ICD-9 or 10 procedure codes and CPT codes, Supplemental Table 3) or repeat lower extremity revascularization ascertained as above. We excluded patients with non-PAD causes of amputation, defined as having an ICD-9 or ICD-10 diagnosis code for trauma, congenital or acquired deformities, or malignancy during the same hospitalization as the lower extremity amputation [13]. Secondary outcomes included amputation and repeat lower extremity revascularization as separate outcomes and death from any cause.

### 2.4. Comorbid Conditions

Demographic variables including age, sex, race and ethnicity, education status, and geographic location were derived at the index date from the CDM database. We defined comorbidities from ICD-9 and ICD-10 diagnosis codes using the Elixhauser Comorbidity software algorithm [14]. Included comorbid conditions are listed in Table 1.

### 2.5. Statistical Analysis

We compared baseline characteristics of patients undergoing endovascular versus surgical revascularization using the count and percentage for categorical variables and the mean and standard deviation for continuous variables and compared the distribution of characteristics between groups using standardized differences [15]. Missing data were imputed by the chained multiple imputation method using all variables listed in Table 1, the outcome of interest, and the Nelson–Aalen estimator [16]. We used inverse probability treatment weighting (IPTW) to reduce selection bias and balance the observed characteristics between the endovascular and surgical revascularization groups [17]. We computed stabilized weights defined as the inverse of the estimated propensity for surgical revascularization calculated from a multivariate logistic regression model and multiplied by a constant equal to the observed proportion of patients with surgical revascularization. We truncated the weights at 0.1 and 10 to improve the IPTW estimator [17]. We used Cox proportional hazard regression to model time to the outcomes of interest and reported adjusted hazard ratios (HRs) and 95% confidence intervals (CIs) using IPTW. Patients were censored for the end of study (Mar 31, 2021), loss of medical coverage, after five years of follow-up, or death, whichever came first. When analyzing death as the outcome of interest, we censored for the end of study, loss of medical coverage, or five years of follow-up, whichever came first.

### 2.6. Statement of Ethics

This study protocol was reviewed and granted an exemption from requiring written informed consent due to the nature of the research; the study protocol was approved by Stanford University Internal Review Board 61, eProtocol #60884.

## 3. Results

We identified 69,395 patients with CKD diagnosed prior to their lower extremity revascularization between January 1, 2013, and January 1, 2021, who met our inclusion criteria (Figure 1). Among patients with CKD, 60,057 (86.5%) underwent an initial strategy of endovascular revascularization and 9,338 (13.5%) underwent surgical revascularization. The majority of endovascular revascularizations occurred in the outpatient setting (70.1%), while the majority of surgical revascularizations occurred in the inpatient setting (89.1%). The mean age of the patients was 72 years, and the cohort was 43.6% female and 61.8% white race. Prior to applying IPTW, patients in the endovascular group had a higher prevalence of diabetes with and without complications and chronic pulmonary disease (Table 1). After applying IPTW, all observed characteristics were balanced between the two treatment groups, as reflected in the maximum absolute standardized differences of <2 (Table 1).

**Figure 2:**
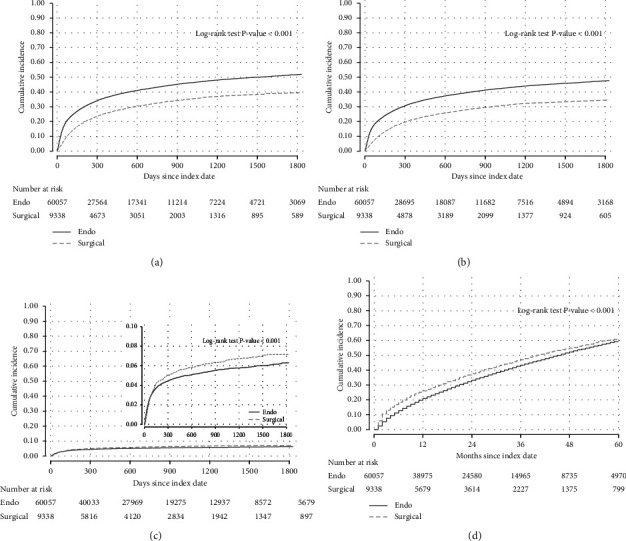
Among patients with chronic kidney disease undergoing an initial endovascular or surgical revascularization, the unadjusted cumulative incidence of (a) major adverse limb events; (b) repeat lower extremity revascularization; (c) lower extremity amputation (the inset shows the same data on an enlarged vertical axis); and (d) death.

During the follow-up period, there were a total of 25,611 MALE, the majority of which were repeat lower extremity revascularizations. The incidence of MALE was higher among patients who underwent endovascular revascularization (incidence 30.1 per 100 person-years) compared with patients who underwent surgical revascularization (incidence 18.7 per 100 person-years) for an adjusted hazard rate of 1.52 (95% CI 1.46−1.59; Table 2, Figure 2(a)). The higher associated risk of MALE was mostly driven by a higher rate of repeat revascularizations in the endovascular group (Table 2, Figure 2(b)). Amputation rates were higher in patients who underwent surgical revascularization (Table 2, Figure 2(c)). The incidence of death from any cause was generally high regardless of revascularization strategy, but patients who underwent endovascular revascularization had a 15% (95% CI 12%−18%) lower adjusted hazard of death (Table 2, Figure 2(d)).

## 4. Discussion

Given the high prevalence of PAD among patients with CKD, the significant morbidity that can result from complications related to PAD, and the paucity of prior trials that focused on patients with CKD [6], we examined the outcomes after endovascular and surgical lower extremity revascularization among a cohort of patients with CKD. We found that endovascular lower extremity revascularization compared with surgical revascularization was associated with a higher adjusted hazard of MALE. More specifically, when we examined MALE outcomes separately, we found that endovascular revascularization was associated with a 65% higher adjusted hazard of repeat revascularization but a 19% lower adjusted hazard of amputation. Patients undergoing endovascular revascularization also had a lower adjusted hazard for death from any cause. We also note that 85% of patients in our cohort underwent an endovascular procedure, rates that are similar to rates seen in the general population [18]. Given past concerns that patients with CKD are less likely to undergo endovascular procedures due to concerns about triggering contrast-induced nephropathy or possibly due to a bias or nihilistic attitude towards patients with CKD (a concept some have termed “renalism” [19]), our findings are reassuring.

The landmark Bypass versus Angioplasty in Severe Ischemia of the Leg (BASIL) trial randomized 452 patients with severe lower limb ischemia due to infrainguinal PAD to receive balloon angioplasty-first versus bypass surgery-first revascularization. In contrast to our findings of lower rates of amputation and death in the endovascular group, in BASIL, there were no significant differences in amputation-free survival or all-cause mortality between the two groups. Differences could stem from differences in the endovascular procedures, since in BASIL, conducted from 1999 to 2004, patients in the endovascular group received balloon angioplasty only, which has been shown to have inferior outcomes when compared with more modern endovascular approaches that may include the use of drug-eluting or covered stents [20].

However, consistent with our results, in BASIL, the endovascular group had a higher rate of repeat revascularization than the surgical group (26% versus 18%, difference of 8%; 95% confidence interval 0.04%−15%) [21]. Our results are also consistent with those of a prior observational study conducted in a Medicare population [22] where patients undergoing endovascular lower extremity revascularization had more repeat revascularization procedures than patients undergoing surgical revascularization and better amputation-free survival. In contrast, a recent study of 12,062 patients who underwent an endovascular-first approach versus 5,166 patients who had surgical bypass for chronic limb-threatening ischemia found no significant differences in amputation-free survival, repeat revascularization, or all-cause mortality between the two treatment approaches [23]. However, that analysis only captured procedures performed during an inpatient hospitalization, which may have selected for patients who were sicker and potentially missed the large number of endovascular procedures that are often performed in an outpatient setting.

Generally, endovascular revascularization is less invasive and carries fewer perioperative risks associated with open surgical techniques such as wound infection and cardiorespiratory complications. These considerations may be particularly important for patients with CKD who often have multiple comorbid conditions and in whom prior studies have confirmed a higher risk of perioperative cardiac and respiratory complications and death [1]. In our analysis, we observed lower rates of death among patients undergoing endovascular revascularization at all time points after the procedure up to five years of follow-up. However, these considerations must be balanced against the higher rates of repeat revascularizations with an endovascular approach, which can carry risks of stent stenosis or thrombosis that are seen less often with surgical approaches. However, technological advances have improved endovascular success rates and clinical outcomes considerably [24], and the lower amputation rates we saw among patients who underwent endovascular revascularization could be a reflection of that improvement. On the other hand, given that current guidelines indicate that patients with more severe tissue loss and disease extent should be considered for surgical bypass [7, 25], patients in the surgical group may already have had a higher risk for requiring amputation (i.e., selection bias).

Our analysis has several strengths, including the diversity of the study cohort in terms of race and ethnicity and that the study cohort was derived from a nationwide database. However, our analysis also has several limitations. First, because our data relied on billing claims, we did not have more granular clinical information such as ankle-brachial indices, severity of symptoms, or vascular anatomy. We were also unable to determine the laterality of the initial revascularization procedure as well as of subsequent repeat revascularization or amputation. It is therefore possible that some of these procedures occurred on the contralateral limb. Second, while the CDM database encompasses a large, diverse population, it only includes patients with private insurance, so our results may not be generalizable to uninsured or to patients with Medicare or Medicaid as their primary insurer. Third, not all patients had laboratory values available, precluding our ability to reliably classify patients into different stages of CKD. We also based our CKD categorization for some patients on the presence of a single laboratory value result, which may have misclassified some patients with transient albuminuria or reductions in kidney function due to acute kidney injury as having CKD. Finally, as noted above, consistent with all observational studies, despite our use of IPTW to balance observed covariates between treatment groups, the lack of randomization leaves open the possibility of residual confounding and bias.

## 5. Conclusions

In summary, in this analysis of patients with CKD undergoing lower extremity revascularization, we found that an endovascular approach was associated with a higher rate of repeated revascularization but a lower risk of subsequent amputation and death compared with surgical revascularization. Our results underscore the importance of integrating patient preferences, surgical risks, and provider expertise to help make decisions about whether to pursue a less invasive approach with fewer upfront risks against the need for future repeat revascularization procedures in patients with CKD. These decisions are especially fraught when considering patients with CKD and PAD, who often also have multiple comorbid conditions. Future clinical trials should deliberately seek to include patients with CKD, who have a high rate of PAD-associated limb complications and who therefore are among the most in need of more definitive guidance.

## Figures and Tables

**Table 1:** Baseline characteristics of patients with chronic kidney disease who received a lower extremity revascularization between January 1, 2013, and January 1, 2021, stratified by type of revascularization.

Variables	Endovascular	Surgical	Maximum absolute standardized difference	
	*N* = 60,057	*N* = 9338	Before IPTW	After IPTW
Mean age (standard deviation), years	72.9 (10.1)	72.3 (10.4)	5.3	0.2
Female sex	26,508 (44.1%)	3747 (40.1%)	8.1	0.4
Race and ethnicity				
Asian	1225 (2.0%)	158 (1.7%)	3.0	0.4
Black	10,665 (17.8%)	1448 (15.5%)	6.5	1.0
Hispanic	8457 (14.1%)	929 (10.0%)	13.3	0.3
White	36,542 (60.9%)	6318 (67.7%)	15.5	1.1
Missing	3168 (5.3%)	485 (5.2%)	—	—
Comorbid conditions				
Coronary artery disease	33,210 (55.3%)	5361 (57.4%)	4.3	1.5
Peripheral artery disease	45,333 (75.5%)	7072 (75.7%)		
Cerebral vascular disease	13,724 (22.9%)	2185 (23.4%)	1.3	1.2
Congestive heart failure	19,996 (33.3%)	3204 (34.3%)	2.1	0.6
Valvular disease	9434 (15.7%)	1804 (19.3%)	9.5	0.3
Hypertension, uncomplicated	52,376 (87.2%)	8065 (86.4%)	2.5	1.1
Hypertension, complicated	30,607 (51.0%)	4450 (47.7%)	6.6	0.9
Pulmonary circulation disorder	2973 (5.0%)	450 (4.8%)	0.6	0.4
Diabetes without chronic complications	32,496 (54.1%)	4368 (46.8%)	14.7	0.7
Diabetes with chronic complications	35,754 (59.5%)	4571 (49.0%)	21.4	0.4
Chronic pulmonary disease	17,974 (29.9%)	3314 (35.5%)	11.9	1.0
Liver disease	2689 (4.5%)	453 (4.9%)	1.8	0.2
Chronic peptic ulcer disease	1082 (1.8%)	218 (2.3%)	3.7	0.5
Neurological	8540 (14.2%)	1306 (14.0%)	0.7	0.4
Hypothyroidism	10,136 (16.9%)	1425 (15.3%)	4.4	0.6
HIV and AIDS	224 (0.4%)	39 (0.4%)	0.7	0.4
Lymphoma	679 (1.1%)	121 (1.3%)	1.5	0.4
Metastatic cancer	1081 (1.8%)	194 (2.1%)	2.0	0.2
Solid tumor without metastasis	5763 (9.6%)	1043 (11.2%)	5.2	0.1
Rheumatoid arthritis/collagen vascular diseases	3552 (5.9%)	512 (5.5%)	1.9	0.1
Obesity	12,713 (21.2%)	1805 (19.3%)	4.6	0.3
Weight loss	5106 (8.5%)	922 (9.9%)	4.8	0.3
Fluid and electrolyte disorders	20,759 (34.6%)	3417 (36.6%)	4.2	0
Coagulation deficiency	6313 (10.5%)	1130 (12.1%)	5.0	1.2
Blood loss anemia	2259 (3.8%)	431 (4.6%)	4.3	0.1
Deficiency anemia	23,182 (38.6%)	3340 (35.8%)	5.9	0.5
Alcohol abuse	1575 (2.6%)	355 (3.8%)	6.7	0.3
Drug abuse	1331 (2.2%)	251 (2.7%)	3.1	0.3
Psychoses	2573 (4.3%)	446 (4.8%)	2.4	0.2
Depression	9424 (15.7%)	1519 (16.3%)	1.6	0.3
Paralysis	3136 (5.2%)	458 (4.9%)	1.4	0.1

All values are *N* (%) except where indicated. Standardized differences are estimated from the maximum absolute difference between the endovascular and surgical covariates for the 5 imputed datasets. IPTW = inverse probability of treatment weighting.

**Table 2:** Median follow-up (in person-years (PY) and incidence of the specified outcomes of interest).

	Endovascular			Surgical			Adjusted HR (95% CI)
	Follow-up, median PY	Events, *N*(%)	Incidence (per 100 PY)	Follow-up, median PY	Events, *N*(%)	Incidence (per 100 PY)	
Major adverse limb events (MALE)	0.7	23,135 (39%)	30.1	0.8	2476 (27%)	18.7	1.52 (1.46–1.59)
Repeat revascularization	0.7	20,917 (35%)	26.3	0.9	2102 (23%)	15.2	1.65 (1.57–1.72)
Amputation	1.5	2896 (5%)	2.5	1.3	486 (5%)	2.9	0.81 (0.73–0.89)
Death	1.4	24,075 (40%)	21.5	1.3	4128 (44%)	24.9	0.85 (0.82–0.88)

Adjusted hazard ratios (HRs) and 95% confidence intervals (CIs) include the inverse probability of treatment weights and compare patients with chronic kidney disease undergoing an initial endovascular versus surgical revascularization.

## Data Availability

This study used data from Optum's de-identifed Clinformatics® Data Mart Database (CDM). Access to these data is controlled by Optum, and inquiries about data access should be directed to them.

## Supplementary Materials

Supplementary Materials Supplemental Table 1: International Classification of Disease, Tenth Edition (ICD-10) procedure codes used to identify lower extremity endovascular revascularizations. Supplemental Table 2: International Classification of Disease, Tenth Edition (ICD-10) procedure codes used to identify lower extremity surgical revascularizations. Supplemental Table 3: International Classification of Disease, Ninth and Tenth edition (ICD-9 and ICD-10) procedure codes and Current Procedural Terminology (CPT) codes used to identify lower extremity amputations.
