# Supplementary material for: Endovascular versus Surgical Lower Extremity Revascularization among Patients with Chronic Kidney Disease
Source: Int J Nephrol. 2023 Dec 16;2023:5586060. doi: 10.1155/2023/5586060 (PMC10748729; doi:10.1155/2023/5586060)
Supplement: Supplementary Materials — Supplemental Table 1: International Classification of Disease, Tenth Edition (ICD-10) procedure codes used to identify lower extremity endovascular revascularizations. Supplemental Table 2: International Classification of Disease, Tenth Edition (ICD-10) procedure codes used to identify lower extremity surgical revascularizations. Supplemental Table 3: International Classification of Disease, Ninth and Tenth edition (ICD-9 and ICD-10) procedure codes and Current Procedural Terminology (CPT) codes used to identify lower extremity amputations. [file 5586060.f1.docx]

**SUPPLEMENTAL MATERIALS**

**Supplemental Table 1: International classification of disease, tenth edition (ICD-10) procedure codes used to identify lower extremity endovascular revascularizations.**

| 0410496 | 041C4J8 | 041D4KB | 041F4KG | 041K49M | 041M4KS | 047C36Z | 047J37Z | 047P3DZ | 047U3EZ | 04BM4ZX | 04RQ4JZ |
| --- | --- | --- | --- | --- | --- | --- | --- | --- | --- | --- | --- |
| 0410497 | 041C4J9 | 041D4KC | 041F4KH | 041J4ZH | 041M4ZL | 047C37Z | 047J3D1 | 047P3EZ | 047U3FZ | 04BM4ZZ | 04RQ4KZ |
| 0410498 | 041C4JB | 041D4KD | 041F4KJ | 041J4ZJ | 041N3JS | 047C3D1 | 047J3DZ | 047P3FZ | 047U3GZ | 04BN3ZX | 04RR47Z |
| 0410499 | 041C4JC | 041D4KF | 041F4KK | 041J4ZK | 041M4ZM | 047C3DZ | 047J3EZ | 047P3GZ | 047U3Z1 | 04BN3ZZ | 04RR4JZ |
| 047C3Z1 | 041C4JD | 041D4KH | 041F4KP | 041J4ZP | 041M4ZP | 047C3FZ | 047J3FZ | 047P3Z1 | 047U3ZZ | 04BN4ZX | 04RR4KZ |
| 041E49D | 041C4JF | 041D4KJ | 041F4KQ | 041J4ZQ | 041M4ZQ | 047C3EZ | 047J3GZ | 047P441 | 047U441 | 04BN4ZZ | 04RS47Z |
| 041049B | 041C4JG | 041D4KK | 041F4Z9 | 041K3JQ | 041M4ZS | 047C3GZ | 047J3Z1 | 047P3ZZ | 047U44Z | 04BP3ZX | 04RS4JZ |
| 041049C | 041C4JH | 041D4KQ | 041F4ZB | 041K3JS | 041N3JQ | 047C3ZZ | 047J3ZZ | 047P44Z | 047U45Z | 04BP3ZZ | 04RS4KZ |
| 041049D | 041C4JJ | 041D4KR | 041F4ZC | 041K49H | 041N49L | 047C441 | 047J441 | 047P45Z | 047U46Z | 04BP4ZX | 04RT47Z |
| 041049F | 041C4JK | 041D4Z0 | 041F4ZD | 041K49J | 041N49M | 047C44Z | 047J44Z | 047P46Z | 047U47Z | 04BP4ZZ | 04RT4JZ |
| 041049G | 041C4JQ | 041D4Z1 | 041H49D | 041K49K | 041N49P | 047C45Z | 047J45Z | 047P4DZ | 047U4D1 | 04BQ3ZX | 04RT4KZ |
| 041049H | 041C4JR | 041D4Z2 | 041F4ZF | 041K49L | 041N49Q | 047C46Z | 047J46Z | 047P47Z | 047U4DZ | 04BQ3ZZ | 04RU47Z |
| 041049J | 041C4K0 | 041E49C | 041F4ZG | 041K49N | 041N49S | 047C47Z | 047J47Z | 047P4D1 | 047U4EZ | 04BQ4ZX | 04RU4JZ |
| 041049K | 041C4K1 | 041D4Z3 | 041F4ZH | 041K49P | 041N4AL | 047C4D1 | 047J4D1 | 047P4GZ | 047U4FZ | 04BQ4ZZ | 04RU4KZ |
| 041049Q | 041C4K2 | 041D4Z4 | 041F4ZJ | 041K49Q | 041N4AM | 047C4DZ | 047J4DZ | 047P4EZ | 047U4GZ | 04BR3ZX | 04RV47Z |
| 041049R | 041C4K3 | 041D4Z5 | 041F4ZK | 041K49S | 041N4AP | 047C4FZ | 047J4EZ | 047P4FZ | 047U4Z1 | 04BR3ZZ | 04RV4JZ |
| 04104A6 | 041C4K4 | 041D4Z6 | 041F4ZP | 041K4AH | 041N4AQ | 047C4EZ | 047J4FZ | 047P4Z1 | 047U4ZZ | 04BR4ZX | 04RV4KZ |
| 04104A7 | 041C4K5 | 041D4Z7 | 041F4ZQ | 041K4AJ | 041N4AS | 047C4GZ | 047J4GZ | 047P4ZZ | 047V341 | 04BR4ZZ | 04RW47Z |
| 04104A8 | 041C4K6 | 041D4Z8 | 041H499 | 041K4AK | 041N4JL | 047C4Z1 | 047J4Z1 | 047Q341 | 047V34Z | 04BS3ZX | 04RW4JZ |
| 04104A9 | 041C4K7 | 041D4Z9 | 041H49B | 041K4AL | 041N4JM | 047C4ZZ | 047J4ZZ | 047Q34Z | 047V35Z | 04BS3ZZ | 04RW4KZ |
| 04104AB | 041C4K9 | 041D4ZB | 041H49C | 041K4AM | 041N4JP | 047D341 | 047K35Z | 047Q35Z | 047V36Z | 04BS4ZX | 04RY47Z |
| 04104AC | 041C4KB | 041D4ZC | 041H49F | 041K4AN | 041N4JQ | 047D34Z | 047K341 | 047Q36Z | 047V37Z | 04BS4ZZ | 04RY4JZ |
| 04104AD | 041C4KC | 041D4ZD | 041H49G | 041K4AP | 041N4JS | 047D35Z | 047K34Z | 047Q37Z | 047V3D1 | 04BT3ZX | 04RY4KZ |
| 04104AF | 041C4KD | 041D4ZF | 041H49H | 041K4AQ | 041N4KL | 047D36Z | 047K36Z | 047Q3D1 | 047V3DZ | 04BT3ZZ |  |
| 04104AG | 041C4KF | 041D4ZG | 041H49J | 041K4AS | 041N4KM | 047D37Z | 047K37Z | 047Q3DZ | 047V3EZ | 04BT4ZX |  |
| 04104AH | 041C4KH | 041D4ZH | 041H49K | 041K4JH | 041N4KP | 047D3D1 | 047K3D1 | 047Q3EZ | 047V3FZ | 04BT4ZZ |  |
| 04104AJ | 041C4KJ | 041D4ZJ | 041H49P | 041K4JJ | 041N4KQ | 047D3DZ | 047K3DZ | 047Q3FZ | 047V3GZ | 04BU3ZX |  |
| 04104AK | 041C4KK | 041D4ZK | 041H49Q | 041K4JK | 041N4ZS | 047D3EZ | 047K3EZ | 047Q3GZ | 047V3Z1 | 04BU3ZZ |  |
| 04104AQ | 041C4KQ | 041D4ZQ | 041H4A9 | 041K4JL | 041N4KS | 047D3GZ | 047K3FZ | 047Q3Z1 | 047V441 | 04BU4ZX |  |
| 04104AR | 041C4KR | 041D4ZR | 041H4AB | 041K4JM | 041N4ZL | 047D3FZ | 047K3GZ | 047Q44Z | 047V44Z | 04BU4ZZ |  |
| 04104J6 | 041C4Z0 | 041E499 | 041H4AC | 041K4JN | 041N4ZM | 047D3Z1 | 047K3Z1 | 047Q441 | 047N37Z | 04CC3ZZ |  |
| 04104J7 | 041D490 | 041E49B | 041H4AD | 041K4JP | 041N4ZP | 047D3ZZ | 047K3ZZ | 047Q46Z | 047Q3ZZ | 04CC4ZZ |  |
| 04104J8 | 041C4Z1 | 041E49F | 041H4AF | 041K4JQ | 041N4ZQ | 047D441 | 047K45Z | 047Q45Z | 047M46Z | 04CD3ZZ |  |
| 04104J9 | 041C4Z2 | 041E49G | 041H4AG | 041K4JS | 041P3JQ | 047D44Z | 047K441 | 047Q4D1 | 047V45Z | 04CD4ZZ |  |
| 04104JB | 041C4Z3 | 041E49H | 041H4AH | 041K4KH | 041P3JS | 047D45Z | 047K44Z | 047Q47Z | 047V46Z | 04CE3ZZ |  |
| 04104JC | 041C4Z4 | 041E49J | 041H4AJ | 041K4KJ | 041P4JQ | 047D46Z | 047K46Z | 047Q4EZ | 047V47Z | 04CE4ZZ |  |
| 04104JD | 041C4Z5 | 041E49K | 041H4AK | 041K4KK | 041P4JS | 047D47Z | 047K47Z | 047Q4DZ | 047V4D1 | 04CF3ZZ |  |
| 04104JF | 041C4Z6 | 041E49P | 041H4AP | 041K4KL | 041Q3JQ | 047D4D1 | 047K4D1 | 047Q4GZ | 047V4DZ | 04CF4ZZ |  |
| 04104JG | 041C4Z7 | 041E49Q | 041H4AQ | 041K4KM | 041Q3JS | 047D4DZ | 047K4DZ | 047Q4FZ | 047V4EZ | 04CH3ZZ |  |
| 04104JH | 041C4Z8 | 041E4A9 | 041H4J9 | 041K4KN | 041Q4JQ | 047D4EZ | 047K4EZ | 047Q4ZZ | 047V4FZ | 04CH4ZZ |  |
| 04104JJ | 041C4Z9 | 041E4AB | 041H4JB | 041K4KP | 041Q4JS | 047D4GZ | 047K4FZ | 047Q4Z1 | 047V4GZ | 04CJ3ZZ |  |
| 04104JK | 041C4ZB | 041E4AC | 041H4JC | 041K4KQ | 041R3JQ | 047D4FZ | 047K4GZ | 047R34Z | 047V4Z1 | 04CJ4ZZ |  |
| 04104JQ | 041C4ZC | 041E4AD | 041H4JD | 041K4KS | 041R3JS | 047D4Z1 | 047K4Z1 | 047R341 | 047W341 | 04CK3ZZ |  |
| 04104JR | 041C4ZD | 041E4AF | 041H4JF | 041K4ZH | 041R4JQ | 047D4ZZ | 047K4ZZ | 047R36Z | 047W34Z | 04CK4ZZ |  |
| 04104K6 | 041C4ZF | 041E4AG | 041H4JG | 041K4ZJ | 041R4JS | 047E341 | 047L341 | 047R35Z | 047W35Z | 04CL3ZZ |  |
| 04104K7 | 041C4ZG | 041E4AH | 041H4JH | 041K4ZK | 041S3JQ | 047E34Z | 047L35Z | 047R3D1 | 047W36Z | 04CL4ZZ |  |
| 04104K8 | 041C4ZH | 041E4AJ | 041H4JJ | 041K4ZL | 041S3JS | 047E35Z | 047L34Z | 047R37Z | 047W37Z | 04CM3ZZ |  |
| 04104K9 | 041C4ZJ | 041E4AK | 041H4JK | 041K4ZM | 041S4JQ | 047E36Z | 047L36Z | 047R3EZ | 047W3D1 | 04CM4ZZ |  |
| 04104KB | 041C4ZK | 041E4AP | 041H4JP | 041L49K | 041S4JS | 047E37Z | 047L37Z | 047R3DZ | 047W3DZ | 04CN3ZZ |  |
| 04104KC | 041C4ZQ | 041E4AQ | 041H4JQ | 041K4ZN | 041T3JQ | 047E3D1 | 047L3D1 | 047R3GZ | 047W3EZ | 04CN4ZZ |  |
| 04104KD | 041C4ZR | 041E4J9 | 041H4K9 | 041K4ZP | 041T3JS | 047E3DZ | 047L3DZ | 047R3FZ | 047W3FZ | 04CP3ZZ |  |
| 04104KF | 041D491 | 041E4JB | 041H4KB | 041K4ZQ | 041T49P | 047E3EZ | 047L3EZ | 047R3ZZ | 047W3GZ | 04CP4ZZ |  |
| 04104KG | 041D492 | 041E4JC | 041H4KC | 041K4ZS | 041T4AQ | 047E3FZ | 047L3FZ | 047R3Z1 | 047W3Z1 | 04CQ3ZZ |  |
| 04104KH | 041D493 | 041E4JD | 041H4KD | 041L3JQ | 041T49Q | 047E3Z1 | 047L3GZ | 047R441 | 047W441 | 04CQ4ZZ |  |
| 04104KJ | 041D494 | 041E4JF | 041H4KF | 041L3JS | 041T49S | 047E3GZ | 047L3Z1 | 047R45Z | 047W44Z | 04CR3ZZ |  |
| 04104KK | 041D495 | 041E4JG | 041H4KH | 041L49H | 041T4AP | 047E3ZZ | 047L3ZZ | 047R44Z | 047W45Z | 04CR4ZZ |  |
| 04104KQ | 041D496 | 041E4JH | 041H4KJ | 041L49J | 041T4AS | 047E441 | 047L441 | 047R47Z | 047W46Z | 04CS3ZZ |  |
| 04104KR | 041D497 | 041E4JJ | 041H4KK | 041L49L | 041T4JP | 047E44Z | 047L45Z | 047R46Z | 047W47Z | 04CS4ZZ |  |
| 04104Z6 | 041D498 | 041E4JK | 041H4KP | 041L49M | 041T4JQ | 047E45Z | 047L44Z | 047R4DZ | 047W4D1 | 04CT3ZZ |  |
| 04104Z7 | 041D499 | 041E4JP | 041H4KQ | 041L49N | 041T4JS | 047E46Z | 047L46Z | 047R4D1 | 047W4DZ | 04CT4ZZ |  |
| 04104Z8 | 041D49B | 041E4JQ | 041H4Z9 | 041L49P | 041T4KP | 047E47Z | 047L47Z | 047R4FZ | 047W4EZ | 04CU3ZZ |  |
| 04104Z9 | 041D49C | 041E4K9 | 041H4ZB | 041L49Q | 041T4KQ | 047E4D1 | 047L4D1 | 047R4EZ | 047W4FZ | 04CU4ZZ |  |
| 04104ZB | 041D49D | 041E4KB | 041H4ZC | 041L49S | 041T4KS | 047E4DZ | 047L4DZ | 047R4Z1 | 047W4GZ | 04CV3ZZ |  |
| 04104ZC | 041D49F | 041E4KC | 041H4ZD | 041L4AH | 041T4ZP | 047E4EZ | 047L4EZ | 047R4GZ | 047W4Z1 | 04CV4ZZ |  |
| 04104ZD | 041D49G | 041E4KD | 041H4ZF | 041L4AJ | 041T4ZQ | 047E4FZ | 047L4FZ | 047S341 | 047Y341 | 04CW3ZZ |  |
| 04104ZF | 041D49H | 041E4KF | 041J49F | 041L4AK | 041T4ZS | 047E4Z1 | 047L4GZ | 047R4ZZ | 047Y34Z | 04CW4ZZ |  |
| 04104ZG | 041D49J | 041E4KH | 041H4ZG | 041L4AL | 041U3JQ | 047E4GZ | 047L4Z1 | 047S35Z | 047Y35Z | 04CY3ZZ |  |
| 04104ZH | 041D49K | 041E4KJ | 041H4ZH | 041L4AM | 041U3JS | 047E4ZZ | 047L4ZZ | 047S34Z | 047Y36Z | 04CY4ZZ |  |
| 04104ZJ | 041D49Q | 041E4KK | 041H4ZJ | 041L4AN | 041U49S | 047F341 | 047M341 | 047S36Z | 047Y37Z | 04PY30Z |  |
| 04104ZK | 041D49R | 041E4KP | 041H4ZK | 041L4AP | 041U49P | 047F34Z | 047M34Z | 047S37Z | 047Y3D1 | 04PY32Z |  |
| 04104ZQ | 041D4A0 | 041E4KQ | 041H4ZP | 041L4AQ | 041U49Q | 047F35Z | 047M36Z | 047S3D1 | 047Y3DZ | 04PY33Z |  |
| 04104ZR | 041D4A1 | 041E4Z9 | 041H4ZQ | 041L4AS | 041U4AP | 047F36Z | 047M35Z | 047S3DZ | 047Y3EZ | 04PY37Z |  |
| 041C490 | 041D4A2 | 041E4ZB | 041J499 | 041L4JH | 041U4AQ | 047F37Z | 047M37Z | 047S3EZ | 047Y3FZ | 04PY3CZ |  |
| 041C491 | 041D4A3 | 041E4ZC | 041J49B | 041L4JJ | 041U4AS | 047F3D1 | 047M3D1 | 047S3FZ | 047Y3GZ | 04PY3DZ |  |
| 041C492 | 041D4A4 | 041E4ZD | 041J49C | 041L4JK | 041U4JP | 047F3DZ | 047M3DZ | 047S3GZ | 047Y3Z1 | 04PY3JZ |  |
| 041C493 | 041D4A5 | 041F49D | 041J49D | 041L4JL | 041U4JQ | 047F3EZ | 047M3EZ | 047S3Z1 | 047Y441 | 04PY3KZ |  |
| 041C494 | 041D4A6 | 041E4ZF | 041J49G | 041L4JM | 041U4JS | 047F3FZ | 047M3FZ | 047S3ZZ | 047Y44Z | 04PY3YZ |  |
| 041C495 | 041D4A7 | 041E4ZG | 041J49H | 041L4JN | 041U4KP | 047F3GZ | 047M3GZ | 047S441 | 047Y45Z | 04PY40Z |  |
| 041C496 | 041D4A8 | 041E4ZH | 041J49J | 041L4JP | 041U4KQ | 047F3ZZ | 047M3Z1 | 047S44Z | 047Y46Z | 04PY42Z |  |
| 041C497 | 041D4A9 | 041E4ZJ | 041J49K | 041L4JQ | 041U4KS | 047F3Z1 | 047M3ZZ | 047S45Z | 047Y47Z | 04PY43Z |  |
| 041C498 | 041D4AB | 041E4ZK | 041J49P | 041L4JS | 041U4ZP | 047F441 | 047M441 | 047S46Z | 047Y4D1 | 04PY47Z |  |
| 041C499 | 041D4AC | 041E4ZP | 041J49Q | 041L4KH | 041U4ZQ | 047F44Z | 047M44Z | 047S47Z | 047Y4DZ | 04PY4CZ |  |
| 041C49B | 041D4AD | 041E4ZQ | 041J4A9 | 041L4KJ | 041U4ZS | 047F45Z | 047M47Z | 047S4D1 | 047Y4EZ | 04PY4DZ |  |
| 041C49C | 041D4AF | 041F499 | 041J4AB | 041L4KK | 041V49P | 047F46Z | 047M45Z | 047S4DZ | 047Y4FZ | 04PY4JZ |  |
| 041C49D | 041D4AG | 041F49B | 041J4AC | 041L4KL | 041V3JQ | 047F47Z | 047M4D1 | 047S4EZ | 047Y4GZ | 04PY4KZ |  |
| 041C49F | 041D4AH | 041F49C | 041J4AD | 041L4KM | 041V3JS | 047F4D1 | 047M4DZ | 047S4FZ | 047Y4Z1 | 04PY4YZ |  |
| 041C49G | 041D4AJ | 041F49F | 041J4AF | 041L4KN | 041V49Q | 047F4DZ | 047M4EZ | 047S4GZ | 04BC3ZX | 04RC47Z |  |
| 041C49H | 041D4AK | 041F49G | 041J4AG | 041L4KP | 041V49S | 047F4EZ | 047M4FZ | 047S4Z1 | 04BC3ZZ | 04RC4JZ |  |
| 041C49J | 041D4AQ | 041F49H | 041J4AH | 041L4KQ | 041V4AP | 047F4FZ | 047M4GZ | 047S4ZZ | 04BC4ZX | 04RC4KZ |  |
| 041C49K | 041D4AR | 041F49J | 041J4AJ | 041L4KS | 041V4AQ | 047F4GZ | 047M4Z1 | 047T341 | 04BC4ZZ | 04RD47Z |  |
| 041C49Q | 041D4J0 | 041F49K | 041J4AK | 041L4ZH | 041V4AS | 047F4ZZ | 047M4ZZ | 047T34Z | 04BD3ZX | 04RD4JZ |  |
| 041C49R | 041D4J1 | 041F49P | 041J4AP | 041L4ZJ | 041V4JP | 047F4Z1 | 047N341 | 047T35Z | 04BD3ZZ | 04RD4KZ |  |
| 041C4A0 | 041D4J2 | 041F49Q | 041J4AQ | 041L4ZK | 041V4JQ | 047H341 | 047N34Z | 047T36Z | 04BD4ZX | 04RE47Z |  |
| 041C4A1 | 041D4J3 | 041F4A9 | 041J4J9 | 041M49L | 041V4JS | 047H34Z | 047N35Z | 047T37Z | 04BD4ZZ | 04RE4JZ |  |
| 041C4A2 | 041D4J4 | 041F4AB | 041J4JB | 041L4ZL | 041V4KP | 047H35Z | 047N3D1 | 047T3D1 | 04BE3ZX | 04RE4KZ |  |
| 041C4A3 | 041D4J5 | 041F4AC | 041J4JC | 041L4ZM | 041V4KQ | 047H36Z | 047N36Z | 047T3DZ | 04BE3ZZ | 04RF47Z |  |
| 041C4A4 | 041D4J6 | 041F4AD | 041J4JD | 041L4ZN | 041V4KS | 047H37Z | 047N3FZ | 047T3EZ | 04BE4ZX | 04RF4JZ |  |
| 041C4A5 | 041D4J7 | 041F4AF | 041J4JF | 041L4ZP | 041V4ZP | 047H3D1 | 047N3DZ | 047T3FZ | 04BE4ZZ | 04RF4KZ |  |
| 041C4A6 | 041D4J8 | 041F4AG | 041J4JG | 041L4ZQ | 041W3JS | 047H3DZ | 047N3EZ | 047T3GZ | 04BF3ZX | 04RH47Z |  |
| 041C4A7 | 041D4J9 | 041F4AH | 041J4JH | 041L4ZS | 041V4ZQ | 047H3EZ | 047N3GZ | 047T3Z1 | 04BF3ZZ | 04RH4JZ |  |
| 041C4A8 | 041D4JB | 041F4AJ | 041J4JJ | 041M3JQ | 041V4ZS | 047H3FZ | 047N3Z1 | 047T3ZZ | 04BF4ZX | 04RH4KZ |  |
| 041C4A9 | 041D4JC | 041F4AK | 041J4JK | 041M3JS | 041W3JQ | 047H3GZ | 047N3ZZ | 047T441 | 04BF4ZZ | 04RJ47Z |  |
| 041C4AB | 041D4JD | 041F4AP | 041J4JP | 041M49M | 041W49P | 047H3Z1 | 047N441 | 047T44Z | 04BH3ZX | 04RJ4JZ |  |
| 041C4AC | 041D4JF | 041F4AQ | 041J4JQ | 041M49P | 041W49Q | 047H3ZZ | 047N44Z | 047T45Z | 04BH3ZZ | 04RJ4KZ |  |
| 041C4AD | 041D4JG | 041F4J9 | 041J4K9 | 041M49Q | 041W49S | 047H441 | 047N45Z | 047T46Z | 04BH4ZX | 04RK47Z |  |
| 041C4AF | 041D4JH | 041F4JB | 041J4KB | 041M49S | 041W4AP | 047H44Z | 047N46Z | 047T47Z | 04BH4ZZ | 04RK4JZ |  |
| 041C4AG | 041D4JJ | 041F4JC | 041J4KC | 041M4AL | 041W4AQ | 047H45Z | 047N47Z | 047T4D1 | 04BJ3ZX | 04RK4KZ |  |
| 041C4AH | 041D4JK | 041F4JD | 041J4KD | 041M4AM | 041W4AS | 047H46Z | 047N4EZ | 047T4DZ | 04BJ3ZZ | 04RL47Z |  |
| 041C4AJ | 041D4JQ | 041F4JF | 041J4KF | 041M4AP | 041W4JP | 047H47Z | 047N4D1 | 047T4EZ | 04BJ4ZX | 04RL4JZ |  |
| 041C4AK | 041D4JR | 041F4JG | 041J4KH | 041M4AQ | 041W4JQ | 047H4D1 | 047N4DZ | 047T4FZ | 04BJ4ZZ | 04RL4KZ |  |
| 041C4AQ | 041D4K0 | 041F4JH | 041J4KJ | 041M4AS | 041W4JS | 047H4DZ | 047N4FZ | 047T4GZ | 04BK3ZX | 04RM47Z |  |
| 041C4AR | 041D4K1 | 041F4JJ | 041J4KK | 041M4JL | 041W4KS | 047H4EZ | 047N4GZ | 047T4Z1 | 04BK3ZZ | 04RM4JZ |  |
| 041C4J0 | 041D4K2 | 041F4JK | 041J4KP | 041M4JM | 041W4KP | 047H4FZ | 047N4Z1 | 047T4ZZ | 04BK4ZX | 04RM4KZ |  |
| 041C4J1 | 041D4K3 | 041F4JP | 041J4KQ | 041M4JP | 041W4KQ | 047H4GZ | 047N4ZZ | 047U341 | 04BK4ZZ | 04RN47Z |  |
| 041C4J2 | 041D4K4 | 041F4JQ | 041J4Z9 | 041M4JQ | 041W4ZP | 047H4Z1 | 047P341 | 047U34Z | 04BL3ZX | 04RN4JZ |  |
| 041C4J3 | 041D4K5 | 041F4K9 | 041J4ZB | 041M4JS | 041W4ZQ | 047J34Z | 047P34Z | 047U35Z | 04BL3ZZ | 04RN4KZ |  |
| 041C4J4 | 041D4K6 | 041F4KB | 041J4ZC | 041M4KL | 041W4ZS | 047H4ZZ | 047P35Z | 047U36Z | 04BL4ZX | 04RP47Z |  |
| 041C4J5 | 041D4K7 | 041F4KC | 041J4ZD | 041M4KM | 047C341 | 047J341 | 047P36Z | 047U37Z | 04BL4ZZ | 04RP4JZ |  |
| 041C4J6 | 041D4K9 | 041F4KD | 041J4ZF | 041M4KP | 047C34Z | 047J35Z | 047P37Z | 047U3D1 | 04BM3ZX | 04RP4KZ |  |
| 041C4J7 |  | 041F4KF | 041J4ZG | 041M4KQ | 047C35Z | 047J36Z | 047P3D1 | 047U3DZ | 04BM3ZZ | 04RQ47Z |  |

**Supplemental Table 2**: International classification of disease, tenth edition (ICD-10) procedure codes used to identify lower extremity surgical revascularizations.

| 0410096 | 041C0A9 | 041D0A9 | 041E0JK | 041H0AP | 041K0AH | 041M0KS | 041W0ZS | 047M041 | 047V041 | 04CJ0ZZ | 04RV0JZ |
| --- | --- | --- | --- | --- | --- | --- | --- | --- | --- | --- | --- |
| 0410097 | 041C0AB | 041D0AB | 041E0JP | 041H0AQ | 041K0AJ | 041M0Op | 047C041 | 047M04Z | 047V04Z | 04CK0Op | 04RV0KZ |
| 0410098 | 041C0AC | 041D0AC | 041E0JQ | 041H0J9 | 041K0AK | 041M0ZL | 047C04Z | 047M05Z | 047V05Z | 04CK0ZZ | 04RV0Op |
| 0410099 | 041C0AD | 041D0AD | 041E0K9 | 041H0JB | 041K0AL | 041M0ZM | 047C05Z | 047M06Z | 047V06Z | 04CL0Op | 04RW07Z |
| 047E041 | 041C0AF | 041D0AF | 041E0KB | 041H0JC | 041K0AM | 041M0ZP | 047C06Z | 047M07Z | 047V07Z | 04CL0ZZ | 04RW0JZ |
| 041E099 | 041C0AG | 041D0AG | 041E0KC | 041H0JD | 041K0AN | 041M0ZQ | 047C07Z | 047M0D1 | 047V0D1 | 04CM0Op | 04RW0KZ |
| 041009B | 041C0AH | 041D0AH | 041E0KD | 041H0JF | 041K0AP | 041M0ZS | 047C0D1 | 047M0DZ | 047V0DZ | 04CM0ZZ | 04RW0Op |
| 041009C | 041C0AJ | 041D0AJ | 041E0KF | 041H0JG | 041K0AQ | 041N09M | 047C0DZ | 047M0EZ | 047V0EZ | 04CN0Op | 04RY07Z |
| 041009D | 041C0AK | 041D0AK | 041E0KG | 041H0JH | 041K0AS | 041N09P | 047C0EZ | 047M0FZ | 047V0FZ | 04CN0ZZ | 04RY0KZ |
| 041009F | 041C0AQ | 041D0AQ | 041E0KH | 041H0JJ | 041K0JH | 041N09Q | 047C0FZ | 047M0GZ | 047V0GZ | 04CP0Op | 04RY0Op |
| 041009G | 041C0AR | 041D0AR | 041E0KJ | 041H0JK | 041K0JJ | 041N09S | 047C0GZ | 047M0Op | 047V0Op | 04CP0ZZ | 047E05Z |
| 041009H | 041C0J0 | 041D0J0 | 041E0KK | 041H0JP | 041K0JK | 041N0AL | 047C0Op | 047M0Z1 | 047V0Z1 | 04CQ0Op | 047H04Z |
| 041009J | 041C0J1 | 041D0J1 | 041E0KP | 041H0JQ | 041K0JL | 041N0AM | 047C0Z1 | 047M0ZZ | 047V0ZZ | 04CQ0ZZ | 047K041 |
| 041009K | 041C0J2 | 041D0J2 | 041E0KQ | 041H0K9 | 041K0JM | 041N0AP | 047C0ZZ | 047N041 | 047W041 | 04CR0Op | 047L0ZZ |
| 041009Q | 041C0J3 | 041D0J3 | 041E0Op | 041H0KB | 041K0JN | 041N0AQ | 047D041 | 047N04Z | 047W04Z | 04CS0Op | 047N0Op |
| 041009R | 041C0J4 | 041D0J4 | 041E0Z9 | 041H0KC | 041K0JP | 041N0AS | 047D04Z | 047N05Z | 047W05Z | 04CS0ZZ | 047Q0Z1 |
| 04100A6 | 041C0J5 | 041D0J5 | 041E0ZB | 041H0KD | 041K0JQ | 041N0JL | 047D05Z | 047N06Z | 047W06Z | 04CT0Op | 047S0GZ |
| 04100A7 | 041C0J6 | 041D0J6 | 041E0ZC | 041H0KF | 041K0JS | 041N0JM | 047D06Z | 047N07Z | 047W07Z | 04CT0ZZ | 047U0FZ |
| 04100A8 | 041C0J7 | 041D0J7 | 041E0ZD | 041H0KG | 041K0KH | 041N0JP | 047D07Z | 047N0D1 | 047W0D1 | 04CU0Op | 047W0EZ |
| 04100A9 | 041C0J8 | 041D0J8 | 041E0ZF | 041H0KH | 041K0KJ | 041N0JQ | 047D0D1 | 047N0DZ | 047W0DZ | 04CU0ZZ | 04BE0Op |
| 04100AB | 041C0J9 | 041D0J9 | 041E0ZG | 041H0KJ | 041K0KK | 041N0JS | 047D0DZ | 047N0EZ | 047W0FZ | 04CV0Op | 04BP0ZZ |
| 04100AC | 041C0JB | 041D0JB | 041E0ZH | 041H0KK | 041K0KL | 041N0KL | 047D0EZ | 047N0FZ | 047W0GZ | 04CV0ZZ | 04BY0Op |
| 04100AD | 041C0JC | 041D0JC | 041E0ZK | 041H0KP | 041K0KM | 041N0KM | 047D0FZ | 047N0GZ | 047W0Op | 04CW0Op | 04CR0ZZ |
| 04100AF | 041C0JD | 041D0JD | 041E0ZP | 041H0KQ | 041K0KN | 041N0KP | 047D0GZ | 047N0Z1 | 047W0Z1 | 04CW0ZZ | 04PY0Op |
| 04100AG | 041C0JF | 041D0JF | 041E0ZQ | 041H0Op | 041K0KP | 041N0KQ | 047D0Op | 047N0ZZ | 047Y041 | 04CY0Op | 04RK07Z |
| 04100AH | 041C0JG | 041D0JG | 041F099 | 041H0Z9 | 041K0KQ | 041N0KS | 047D0Z1 | 047P041 | 047Y04Z | 04CY0ZZ | 04RR0JZ |
| 04100AJ | 041C0JH | 041D0JH | 041F09B | 041H0ZB | 041K0KS | 041N0Op | 047D0ZZ | 047P04Z | 047Y05Z | 04JY0Op |  |
| 04100AK | 041C0JJ | 041D0JJ | 041F09C | 041H0ZC | 041K0Op | 041N0ZL | 047E04Z | 047P05Z | 047Y06Z | 04JY0ZN |  |
| 04100AQ | 041C0JK | 041D0JK | 041F09D | 041H0ZD | 041K0ZH | 041N0ZM | 047E06Z | 047P06Z | 047Y07Z | 04PY00Z |  |
| 04100AR | 041C0JQ | 041D0JQ | 041F09F | 041H0ZF | 041K0ZJ | 041N0ZP | 047E07Z | 047P07Z | 047Y0D1 | 04PY02Z |  |
| 04100J6 | 041C0JR | 041D0JR | 041F09G | 041H0ZG | 041K0ZK | 041N0ZQ | 047E0D1 | 047P0D1 | 047Y0DZ | 04PY03Z |  |
| 04100J7 | 041C0K0 | 041D0K0 | 041F09H | 041H0ZJ | 041K0ZL | 041N0ZS | 047E0DZ | 047P0DZ | 047Y0EZ | 04PY07Z |  |
| 04100J8 | 041C0K1 | 041D0K1 | 041F09J | 041H0ZK | 041K0ZM | 041P0JQ | 047E0EZ | 047P0EZ | 047Y0FZ | 04PY0CZ |  |
| 04100J9 | 041C0K2 | 041D0K2 | 041F09K | 041H0ZP | 041K0ZN | 041P0JS | 047E0FZ | 047P0FZ | 047Y0GZ | 04PY0DZ |  |
| 04100JB | 041C0K3 | 041D0K3 | 041F09P | 041H0ZQ | 041K0ZQ | 041Q0JQ | 047E0GZ | 047P0GZ | 047Y0Op | 04PY0JZ |  |
| 04100JC | 041C0K4 | 041D0K4 | 041F09Q | 041J099 | 041K0ZS | 041Q0JS | 047E0Op | 047P0Op | 047Y0Z1 | 04PY0KZ |  |
| 04100JD | 041C0K5 | 041D0K5 | 041F0A9 | 041J09B | 041L09H | 041Q0Op | 047E0Z1 | 047P0Z1 | 04BC0Op | 04PY0YZ |  |
| 04100JF | 041C0K6 | 041D0K6 | 041F0AB | 041J09C | 041L09J | 041R0JQ | 047E0ZZ | 047P0ZZ | 04BC0ZX | 04RC07Z |  |
| 04100JG | 041C0K7 | 041D0K7 | 041F0AC | 041J09D | 041L09K | 041R0Op | 047F041 | 047Q041 | 04BC0ZZ | 04RC0JZ |  |
| 04100JH | 041C0K8 | 041D0K8 | 041F0AD | 041J09F | 041L09L | 041S0JQ | 047F04Z | 047Q04Z | 04BD0Op | 04RC0KZ |  |
| 04100JJ | 041C0K9 | 041D0K9 | 041F0AF | 041J09G | 041L09M | 041S0JS | 047F05Z | 047Q05Z | 04BD0ZX | 04RC0Op |  |
| 04100JK | 041C0KB | 041D0KB | 041F0AG | 041J09H | 041L09N | 041S0Op | 047F06Z | 047Q06Z | 04BD0ZZ | 04RD07Z |  |
| 04100JQ | 041C0KC | 041D0KC | 041F0AH | 041J09J | 041L09P | 041T09Q | 047F07Z | 047Q07Z | 04BE0ZX | 04RD0JZ |  |
| 04100JR | 041C0KD | 041D0KD | 041F0AJ | 041J09K | 041L09Q | 041T09S | 047F0D1 | 047Q0D1 | 04BE0ZZ | 04RD0KZ |  |
| 04100K6 | 041C0KF | 041D0KF | 041F0AK | 041J09P | 041L09S | 041T0AP | 047F0DZ | 047Q0DZ | 04BF0Op | 04RD0Op |  |
| 04100K7 | 041C0KG | 041D0KG | 041F0AP | 041J09Q | 041L0AH | 041T0AQ | 047F0EZ | 047Q0EZ | 04BF0ZX | 04RE07Z |  |
| 04100K8 | 041C0KH | 041D0KH | 041F0AQ | 041J0A9 | 041L0AJ | 041T0JP | 047F0FZ | 047Q0FZ | 04BF0ZZ | 04RE0JZ |  |
| 04100K9 | 041C0KJ | 041D0KJ | 041F0J9 | 041J0AB | 041L0AK | 041T0JQ | 047F0GZ | 047Q0GZ | 04BH0Op | 04RE0KZ |  |
| 04100KB | 041C0KK | 041D0KK | 041F0JB | 041J0AC | 041L0AL | 041T0JS | 047F0Op | 047Q0Op | 04BH0ZX | 04RE0Op |  |
| 04100KC | 041C0KQ | 041D0KQ | 041F0JC | 041J0AD | 041L0AM | 041T0KP | 047F0Z1 | 047Q0ZZ | 04BH0ZZ | 04RF07Z |  |
| 04100KD | 041C0KR | 041D0KR | 041F0JD | 041J0AF | 041L0AN | 041T0KS | 047F0ZZ | 047R041 | 04BJ0Op | 04RF0JZ |  |
| 04100KF | 041C0Op | 041D0Op | 041F0JF | 041J0AG | 041L0AP | 041T0Op | 047H041 | 047R04Z | 04BJ0ZX | 04RF0KZ |  |
| 04100KG | 041C0Z0 | 041D0Z0 | 041F0JG | 041J0AH | 041L0AQ | 041T0ZP | 047H05Z | 047R05Z | 04BJ0ZZ | 04RF0Op |  |
| 04100KH | 041C0Z1 | 041D0Z1 | 041F0JH | 041J0AJ | 041L0AS | 041T0ZQ | 047H06Z | 047R06Z | 04BK0Op | 04RH07Z |  |
| 04100KJ | 041C0Z2 | 041D0Z2 | 041F0JJ | 041J0AK | 041L0JH | 041T0ZS | 047H07Z | 047R07Z | 04BK0ZX | 04RH0JZ |  |
| 04100KK | 041C0Z3 | 041D0Z3 | 041F0JK | 041J0AP | 041L0JJ | 041U09P | 047H0D1 | 047R0D1 | 04BK0ZZ | 04RH0KZ |  |
| 04100KQ | 041C0Z4 | 041D0Z4 | 041F0JP | 041J0AQ | 041L0JK | 041U09Q | 047H0DZ | 047R0DZ | 04BL0Op | 04RH0Op |  |
| 04100KR | 041C0Z5 | 041D0Z5 | 041F0JQ | 041J0J9 | 041L0JL | 041U09S | 047H0EZ | 047R0EZ | 04BL0ZX | 04RJ07Z |  |
| 04100Z6 | 041C0Z6 | 041D0Z6 | 041F0K9 | 041J0JB | 041L0JM | 041U0AP | 047H0FZ | 047R0FZ | 04BL0ZZ | 04RJ0JZ |  |
| 04100Z7 | 041C0Z7 | 041D0Z7 | 041F0KB | 041J0JC | 041L0JN | 041U0AQ | 047H0GZ | 047R0GZ | 04BM0Op | 04RJ0KZ |  |
| 04100Z8 | 041C0Z8 | 041D0Z8 | 041F0KC | 041J0JD | 041L0JP | 041U0AS | 047H0Op | 047R0Op | 04BM0ZX | 04RJ0Op |  |
| 04100Z9 | 041C0Z9 | 041D0Z9 | 041F0KD | 041J0JF | 041L0JQ | 041U0JP | 047H0Z1 | 047R0Z1 | 04BM0ZZ | 04RK0JZ |  |
| 04100ZB | 041C0ZB | 041D0ZB | 041F0KF | 041J0JG | 041L0JS | 041U0JQ | 047H0ZZ | 047R0ZZ | 04BN0Op | 04RK0KZ |  |
| 04100ZC | 041C0ZC | 041D0ZC | 041F0KG | 041J0JH | 041L0KH | 041U0JS | 047J041 | 047S041 | 04BN0ZX | 04RK0Op |  |
| 04100ZD | 041C0ZD | 041D0ZD | 041F0KH | 041J0JJ | 041L0KJ | 041U0KP | 047J04Z | 047S04Z | 04BN0ZZ | 04RL07Z |  |
| 04100ZF | 041C0ZF | 041D0ZF | 041F0KJ | 041J0JK | 041L0KK | 041U0KQ | 047J05Z | 047S05Z | 04BP0Op | 04RL0JZ |  |
| 04100ZG | 041C0ZG | 041D0ZG | 041F0KK | 041J0JP | 041L0KL | 041U0KS | 047J06Z | 047S06Z | 04BP0ZX | 04RL0KZ |  |
| 04100ZH | 041C0ZH | 041D0ZH | 041F0KP | 041J0JQ | 041L0KM | 041U0Op | 047J07Z | 047S07Z | 04BQ0Op | 04RL0Op |  |
| 04100ZJ | 041C0ZJ | 041D0ZJ | 041F0KQ | 041J0K9 | 041L0KN | 041U0ZQ | 047J0D1 | 047S0D1 | 04BQ0ZX | 04RM07Z |  |
| 04100ZK | 041C0ZK | 041D0ZQ | 041F0Op | 041J0KB | 041L0KP | 041U0ZS | 047J0DZ | 047S0DZ | 04BQ0ZZ | 04RM0JZ |  |
| 04100ZQ | 041C0ZQ | 041D0ZR | 041F0Z9 | 041J0KC | 041L0KQ | 041V09P | 047J0EZ | 047S0EZ | 04BR0Op | 04RM0KZ |  |
| 04100ZR | 041D090 | 041E09B | 041F0ZB | 041J0KD | 041L0KS | 041V09Q | 047J0FZ | 047S0FZ | 04BR0ZX | 04RM0Op |  |
| 041C091 | 041D091 | 041E09C | 041F0ZC | 041J0KF | 041L0Op | 041V09S | 047J0GZ | 047S0Op | 04BR0ZZ | 04RN07Z |  |
| 041C092 | 041D092 | 041E09D | 041F0ZD | 041J0KG | 041L0ZH | 041V0AP | 047J0Op | 047S0Z1 | 04BS0Op | 04RN0JZ |  |
| 041C093 | 041D093 | 041E09F | 041F0ZF | 041J0KH | 041L0ZJ | 041V0AQ | 047J0Z1 | 047S0ZZ | 04BS0ZX | 04RN0KZ |  |
| 041C094 | 041D094 | 041E09G | 041F0ZG | 041J0KJ | 041L0ZK | 041V0AS | 047J0ZZ | 047T041 | 04BS0ZZ | 04RN0Op |  |
| 041C095 | 041D095 | 041E09H | 041F0ZH | 041J0KK | 041L0ZL | 041V0JP | 047K04Z | 047T04Z | 04BT0Op | 04RP07Z |  |
| 041C096 | 041D096 | 041E09J | 041F0ZK | 041J0KP | 041L0ZM | 041V0JQ | 047K05Z | 047T05Z | 04BT0ZX | 04RP0JZ |  |
| 041C097 | 041D097 | 041E09K | 041F0ZP | 041J0KQ | 041L0ZN | 041V0JS | 047K06Z | 047T06Z | 04BT0ZZ | 04RP0KZ |  |
| 041C098 | 041D098 | 041E09P | 041F0ZQ | 041J0Op | 041L0ZP | 041V0KP | 047K07Z | 047T07Z | 04BU0Op | 04RP0Op |  |
| 041C099 | 041D099 | 041E09Q | 041H099 | 041J0Z9 | 041L0ZQ | 041V0KQ | 047K0D1 | 047T0D1 | 04BU0ZX | 04RQ07Z |  |
| 041C09B | 041D09B | 041E0A9 | 041H09B | 041J0ZB | 041M09L | 041V0KS | 047K0DZ | 047T0DZ | 04BU0ZZ | 04RQ0JZ |  |
| 041C09C | 041D09C | 041E0AB | 041H09C | 041J0ZC | 041M09M | 041V0Op | 047K0EZ | 047T0EZ | 04BV0Op | 04RQ0KZ |  |
| 041C09D | 041D09D | 041E0AC | 041H09D | 041J0ZD | 041M09P | 041V0ZP | 047K0FZ | 047T0FZ | 04BV0ZX | 04RQ0Op |  |
| 041C09F | 041D09F | 041E0AD | 041H09F | 041J0ZF | 041M09Q | 041V0ZQ | 047K0GZ | 047T0GZ | 04BV0ZZ | 04RR07Z |  |
| 041C09G | 041D09G | 041E0AF | 041H09G | 041J0ZG | 041M09S | 041W09P | 047K0Op | 047T0Op | 04BW0Op | 04RR0KZ |  |
| 041C09H | 041D09H | 041E0AG | 041H09H | 041J0ZH | 041M0AL | 041W09Q | 047K0Z1 | 047T0Z1 | 04BW0ZX | 04RR0Op |  |
| 041C09J | 041D09J | 041E0AH | 041H09J | 041J0ZJ | 041M0AM | 041W09S | 047K0ZZ | 047T0ZZ | 04BW0ZZ | 04RS07Z |  |
| 041C09K | 041D09K | 041E0AJ | 041H09K | 041J0ZK | 041M0AP | 041W0AP | 047L041 | 047U041 | 04BY0ZN | 04RS0JZ |  |
| 041C09Q | 041D09Q | 041E0AK | 041H09P | 041J0ZP | 041M0AQ | 041W0AQ | 047L04Z | 047U04Z | 04CC0Op | 04RS0KZ |  |
| 041C09R | 041D09R | 041E0AP | 041H09Q | 041J0ZQ | 041M0AS | 041W0AS | 047L05Z | 047U05Z | 04CC0ZZ | 04RS0Op |  |
| 041C0A0 | 041D0A0 | 041E0AQ | 041H0A9 | 041K09H | 041M0JL | 041W0JP | 047L06Z | 047U06Z | 04CD0Op | 04RT07Z |  |
| 041C0A1 | 041D0A1 | 041E0J9 | 041H0AB | 041K09J | 041M0JM | 041W0JQ | 047L07Z | 047U07Z | 04CD0ZZ | 04RT0JZ |  |
| 041C0A2 | 041D0A2 | 041E0JB | 041H0AC | 041K09K | 041M0JP | 041W0JS | 047L0D1 | 047U0D1 | 04CE0Op | 04RT0KZ |  |
| 041C0A3 | 041D0A3 | 041E0JC | 041H0AD | 041K09L | 041M0JQ | 041W0KP | 047L0DZ | 047U0DZ | 04CE0ZZ | 04RT0Op |  |
| 041C0A4 | 041D0A4 | 041E0JD | 041H0AF | 041K09M | 041M0JS | 041W0KQ | 047L0EZ | 047U0EZ | 04CF0Op | 04RU07Z |  |
| 041C0A5 | 041D0A5 | 041E0JF | 041H0AG | 041K09N | 041M0KL | 041W0KS | 047L0FZ | 047U0GZ | 04CF0ZZ | 04RU0JZ |  |
| 041C0A6 | 041D0A6 | 041E0JG | 041H0AH | 041K09P | 041M0KM | 041W0Op | 047L0GZ | 047U0Op | 04CH0Op | 04RU0KZ |  |
| 041C0A7 | 041D0A7 | 041E0JH | 041H0AJ | 041K09Q | 041M0KP | 041W0ZP | 047L0Op | 047U0Z1 | 04CH0ZZ | 04RU0Op |  |
| 041C0A8 | 041D0A8 | 041E0JJ | 041H0AK | 041K09S | 041M0KQ | 041W0ZQ | 047L0Z1 | 047U0ZZ | 04CJ0Op | 04RV07Z |  |

**Supplemental Table 3**: International Classification of Disease, Ninth and Tenth edition (ICD-9, ICD-10) procedure codes and Current Procedural Terminology (CPT) codes used to identify lower extremity amputations.

|  | **ICD-9** | **ICD-10** | **CPT** |
| --- | --- | --- | --- |
| **Above knee amputation** | 84.16 84.17 84.18 84.19 | \| 0Y6F0ZZ \| 0Y6D0Z2 \| 0Y6C0Z2 \| 0Y640ZZ \| 0Y6C0Z3 \| \| --- \| --- \| --- \| --- \| --- \| \| 0Y6G0ZZ \| 0Y6D0Z3 \| 0Y680ZZ \| 0Y620ZZ \|  \| \| 0Y6C0Z1 \| 0Y670ZZ \| 0Y6D0Z1 \| 0Y630ZZ \|  \| | 27295, 27590-92 27598 |
| **Below knee amputation** | 84.13 84.14 84.15 | \| 0Y6H0Z3 \| 0Y6H0Z2 \| 0Y6J0Z3 \| 0Y6J0Z1 \| 0Y6H0Z1 \| \| --- \| --- \| --- \| --- \| --- \| \| 0Y6J0Z2 \|  \|  \|  \|  \| | 27880-82, 27888-89 |
| **Below ankle amputation** | 84.11 84.12 | \| 0Y6P0Z0 \| 0Y6Q0Z1 \| 0Y6R0Z2 \| 0Y6M0Z8 \| \| --- \| --- \| --- \| --- \| \| 0Y6P0Z1 \| 0Y6Q0Z2 \| 0Y6R0Z3 \| 0Y6M0Z9 \| \| 0Y6P0Z2 \| 0Y6Q0Z3 \| 0Y6S0Z0 \| 0Y6M0ZB \| \| 0Y6P0Z3 \| 0Y6R0Z0 \| 0Y6S0Z1 \| 0Y6M0ZC \| \| 0Y6Q0Z0 \| 0Y6R0Z1 \| 0Y6S0Z2 \| 0Y6M0ZD \| \| 0Y6S0Z3 \| 0Y6T0Z3 \| 0Y6U0Z3 \| 0Y6M0ZF \| \| 0Y6T0Z0 \| 0Y6U0Z0 \| 0Y6V0Z0 \| 0Y6N0Z4 \| \| 0Y6T0Z1 \| 0Y6U0Z1 \| 0Y6V0Z1 \| 0Y6N0Z5 \| \| 0Y6T0Z2 \| 0Y6U0Z2 \| 0Y6V0Z3 \| 0Y6N0Z6 \| \| 0Y6V0Z2 \| 0Y6W0Z1 \| 0Y6W0Z3 \| 0Y6N0Z7 \| \| 0Y6W0Z0 \| 0Y6W0Z2 \| 0Y6X0Z0 \| 0Y6N0Z9 \| \| 0Y6X0Z1 \| 0Y6X0Z2 \| 0Y6X0Z3 \| 0Y6N0ZB \| \| 0Y6Y0Z0 \| 0Y6Y0Z1 \| 0Y6Y0Z2 \| 0Y6N0ZC \| \| 0Y6Y0Z3 \| 0Y6M0Z4 \| 0Y6M0Z5 \| 0Y6N0ZD \| \| 0Y6M0Z6 \| 0Y6M0Z7 \| 0Y6N0Z8 \| 0Y6N0ZF \| \| 0Y6M0Z0 \| 0Y6N0Z0 \|  \|  \| | 28800, 28805 |
